# Alterations in the airborne bacterial community during Asian dust events occurring between February and March 2015 in South Korea

**DOI:** 10.1038/srep37271

**Published:** 2016-11-16

**Authors:** Seho Cha, Dongwook Lee, Jun Hyeong Jang, Sora Lim, Dahye Yang, Taegun Seo

**Affiliations:** 1 Department of Life Science, Dongguk University-Seoul, Goyang, 10326, South Korea

## Abstract

During Asian dust events, a relatively high concentration of particulate matter is transported by wind from arid and semi-arid regions, such as the Gobi and Taklamakan deserts, to nearby countries, including China, Korea, and Japan. The dust particles contain various microorganisms, which can affect human health as well as the environmental microbe population. In the current study, we investigated the characteristics of the airborne bacterial community during Asian dust events between February and March 2015 in South Korea. Bacterial diversity indexes such as operational taxonomic units, Chao1 and Inverse Simpson index were increased, along with total 16S rRNA gene copy number during Asian dust events. The bacterial community structure during Asian dust events was clearly distinguishable from that during non-Asian dust days. The genera *Bacillus* and *Modestobacter* were increased 3.9- and 2.7-fold, respectively, while *Escherichia-Shigella* was decreased by 89.8%. A non-metric multidimensional scaling plot with metadata analysis revealed association of particulate matter concentration, but not temperature, humidity or wind speed, with bacterial community structure, suggesting that the newly transported dust particles contain various microorganisms that influence the airborne bacterial environment.

Asian dust (AD) events represent a major environmental issue in East Asia, since they cause various environmental and industrial problems and influence human health^1^,^2^,^3^,^4^. During AD events, dust particles that contain various microorganisms and heavy metals are transported over long distances by the wind, leading to alterations in the airborne environment^5^,^6^. Owing to increasing desertification of the Gobi and Taklamakan desert regions along with accelerating industrialization, the occurrence of AD events is becoming more frequent. According to reports from Korea meteorological administration (KMA), the average length of an AD event has increased from 3.3 days in the 1990 s to 8.6 days in the 2000 s. In addition, AD events most often occur in the spring, especially between March and May, leading to concerns about seasonal issues^7^,^8^. However, it is important to note that these events are not limited to spring. A total of 29 days of AD events have been recorded during winter months from December to February, in Seoul, Korea, since 2000 (KMA database).

Studies on the relationship between AD and human health, in particular, respiratory diseases and skin irritation, have focused on the chemical composition, size, and amount of transported dust particles^2^,^9^,^10^,^11^,^12^. Small-sized dust particles can cause inflammatory responses in the respiratory tract or lung region^13^,^14^, and microorganisms within dust particles act synergistically to induce inflammatory mechanisms^15^,^16^. He *et al.*^17^ showed that while helper T-cell type 2-associated cytokines are activated by both original and heat-treated AD particles at 360 °C for 30 min, mRNA levels of Toll-like receptor 2 (TLR2) and NACHT, LRR and PYD domain-containing protein 3 (NALP3) are increased by original AD particles, suggesting that microbial materials participate in several inflammation-related mechanisms.

Earlier findings mentioned above support the possibility that microorganism constituents of dust particles trigger respiratory diseases or allergic responses in humans. In view of the significant increase in the number of microorganisms in the airborne environment during AD events^18^,^19^, elucidation of the biological characteristics of these particles, including identification of newly transported microorganisms, diversity of communities, and proportions of specific taxa, is critical for human health. However, relatively limited studies have focused on determining the bacterial characteristics of airborne environments during AD episodes owing to a number of environmental and technical problems. (i) Since AD events occur over only a few days every year, it is difficult to collect sufficient samples to perform effective statistical analysis. (ii) There are no arrangements in place to collect airborne samples for elucidation of biological features on ordinary days. (iii) The existing culture and PCR-based methods for analyzing bacterial structures, including the plate method, Sanger sequencing, and DGGE, are not suitable for handling unculturable bacteria and multiple data on a large scale^18^,^20^,^21^.

In the current study, we monitored and collected airborne dust particles from January to March 2015 in South Korea, and obtained daily samples, including 9 days of AD events and 11 non-AD days. Fortunately, AD events were relatively frequent during this period, allowing us to secure sufficient samples for comparison with normal dust-free days. The whole metagenomics approach using next-generation sequencing with the Roche 454 system was applied for analysis of more than 200,000 sequences.

The main aim of this study was to determine the bacterial community structures of the airborne environment during AD events in Korea and analyze the information obtained using a metagenomic approach. Establishment of bacterial characteristics through continuous monitoring of the airborne environment during AD events may aid in understanding the biological features of dust particles as well as management of associated environmental impacts and human health problems.

## Results

### Environmental conditions and bacterial diversity analysis

**Figure 1:**
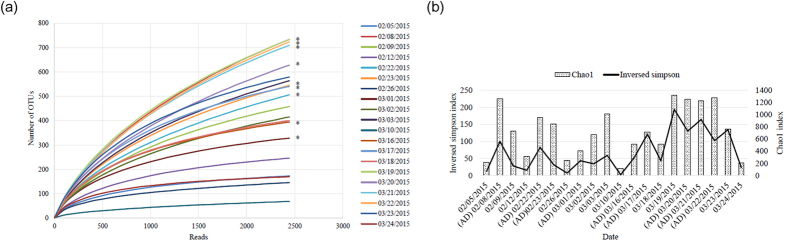
Alpha diversity analysis during Asian dust (AD) events and non-AD periods. (**a**) Operational taxonomic unit analysis of the collected samples at the species level. Asterisks indicate Asian dust samples. (**b**) Chao1 and inverse Simpson index of the collected samples at the species level.

To determine the airborne bacterial communities during AD events, we collected dust samples using a high-volume sampler from January to March 2015 from the rooftop of the university building in Goyang-si, South Korea. During the sampling period, no precipitation occurred to limit the collection of dust particles. The humidity of the sampling period varied between 26.6–71.3%, temperature from −9.9 to 10.4 °C, and wind speed from 1 to 4.5 m/s. From February to March 2015, nine days of AD events samples were collected. We examined samples from a total of 20 days, including 11 non-AD days. As shown in Supplementary Table S1. Using the classification data, we confirmed operational taxonomic units (OTUs) at the species level during both AD event and non-AD days. As shown in Fig. 1a and Supplementary Table S1, OTU results varied from around 68 (March 10, non-AD) to 733 (February 8, AD; March 19, non-AD), with the average value for AD samples being significantly higher than that for non-AD samples (P-value = 0.019, independent t-test). In addition, the Chao1 index, but not inverse Simpson index (representing richness and evenness diversity of the bacterial community, respectively) was significantly different between AD and non-AD samples (Fig. 1b and Supplementary Table S1, P-value = 0.029, independent t-test). These results suggest that airborne bacterial diversity (at least the richness index) is increased during AD events, and transported dust particles contain various microorganisms that can alter an airborne bacterial community.

### 16S rRNA gene copy numbers in the collected samples

**Figure 2:**
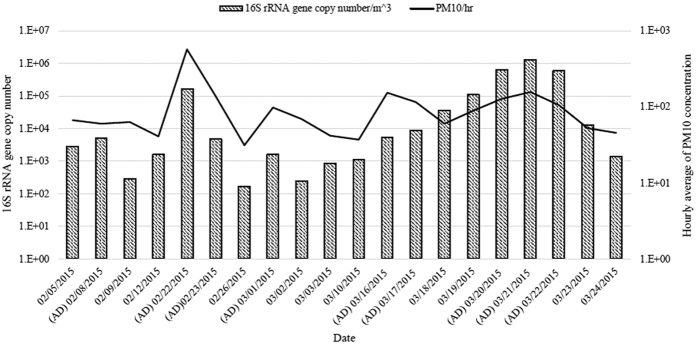
Relationship between the 16S rRNA gene copy number and PM10 concentration during AD events and non-AD periods. Spearman correlation coefficient between the two elements is 0.6496 and p-value is 0.00247.

Since newly transported dust particles during AD events were shown to contain various bacteria that could influence airborne bacterial diversity, we measured the 16S rRNA gene copy number in samples to estimate the relative amounts of different bacterial communities in the airborne environment. For quantification, *E. coli* ATCC 11775 was used as the standard strain containing one copy of the 16S rRNA gene in the genome. Using the standard curve of real-time PCR results based on the *E. coli* strain, the 16S rRNA gene copy number was determined in samples during both AD events and non-AD days. During AD events, a relatively higher concentration of particulate matter 10 (PM10), presenting dust particles under 10 μm in size, was observed in the airborne environment. As expected, the 16S rRNA gene copy number was correlated with PM10 concentration (Spearman correlation coefficient, 0.6496; P-value = 0.00247), and both factors were significantly higher in AD samples, compared to non-AD samples (independent t-test, P-value = 0.047 and 0.027, respectively) (Fig. 2). In particular, the average 16S rRNA gene copy number was 20 times higher in AD event samples, with the highest 16S rRNA gene copy number (AD sample, March 21) being ~8,000 times greater than the lowest 16S rRNA gene copy number in a non-AD sample (February 26). Together with diversity index data, these results support an increase in the number of microbes transported with dust particles during AD events, which can influence the local airborne bacterial community.

### Non-metric Multidimensional Scaling (NMDS) analysis

**Figure 3:**
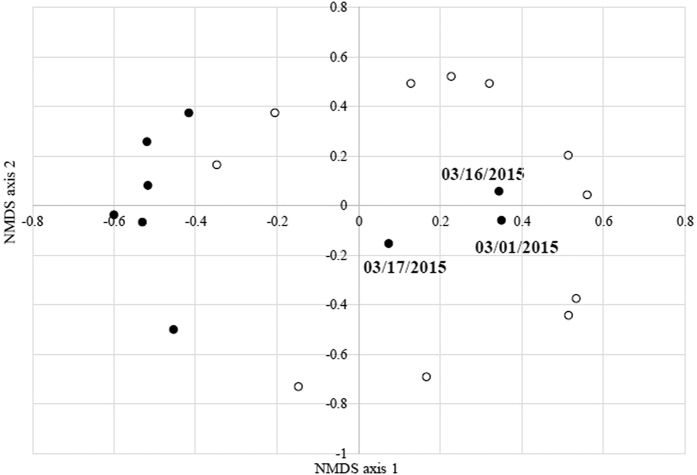
Nonmetric multidimensional scaling plot. Closed circles indicate the days of AD events, and open circles indicate the non-AD days.

To elucidate whether characteristics of the bacterial community are clearly distinguishable based on transported microorganisms during AD events, we analysed the differences in bacterial community structures between AD and non-AD days using non-metric multidimensional scaling (NMDS) at the species level. Among the nine days of AD events, samples from six days are presented along the left direction of axis1, and samples from most days of non-AD days presented along the right direction of axis1 (Fig. 3). We observed no differences between AD and non-AD samples along axis2. The bacterial community structures of dust collected from several AD events (March 1, 16, and 17) were presented in the right direction of axis1 with non-AD days. The results obtained for three days of AD events were correlated with the Chao1 diversity index, which presented a relatively low level of diversity, compared to other AD samples. The differential distribution of bacterial structures between AD and non-AD samples on the NMDS plot was statistically confirmed using analysis of molecular variance (AMOVA) (P-value = 0.026). To further determine the specific factors affecting distribution on the NMDS plot, we calculated the correlation coefficient between the direction of axis and experimental or environmental factors, including 16S rRNA gene copy number, PM10 concentration, temperature, wind speed, and humidity. As shown in Table 1, only two factors (16S rRNA gene copy number and PM10 concentration) were significantly correlated with the negative direction along axis1 of NMDS plots (Correlation coefficient = 0.55 and 0.46; P-value = 0.0069 and 0.029). These results suggest that the airborne bacterial community structure was altered through influx of newly transported microorganisms, but not by general meteorological conditions such as temperature, wind speed, and humidity, during the AD events under investigation. Construction of a phylogenetic tree with the ThetaYC algorithm at the species level additionally revealed considerably different bacterial community structures of AD and non-AD samples (Supplementary Figure S1, both weighted and unweighted significance values between AD and non-AD days were less than 0.05). Our data indicate that the airborne bacterial community is clearly distinguishable between AD and non-AD episodes owing to newly transported bacteria, with no contribution from meteorological elements. Although the airborne microbial environment was gradually restored through atmospheric flow within a few days, continuously occurring AD events and the consequent transient increase in microorganisms may contribute to several human diseases and exert widespread effects on the ecological system.

### Analysis of the bacterial community during AD and non-AD periods at the phylum level

**Figure 4:**
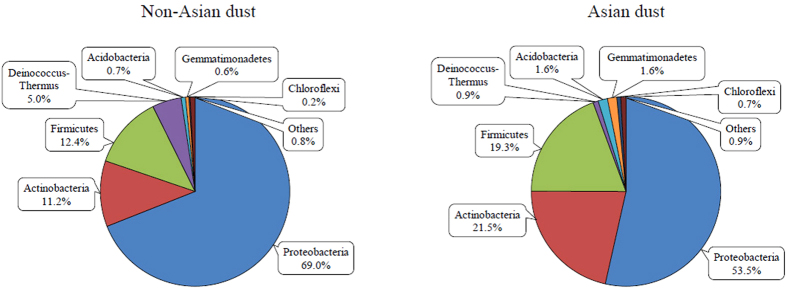
Bacterial community at the phylum level. Within the total percentage, phyla with >0.5% abundance are presented.

To identify the bacterial community in the airborne environment between February and March 2015, pyrosequencing results were classified using Mothur software with the SILVA database^22^,^23^. During the non-AD period, the predominant phylum in the airborne environment was Proteobacteria, followed by Firmicutes, Actinobacteria, Deinococcus-Thermus, Acidobacteria, Gemmatimonadetes, and Chloroflexi (Fig. 4). Proteobacteria remained the dominant phylum during AD events, but the proportion decreased from 69% to 53.5%. Notably, the relative amounts of several other phyla, including Actinobacteria, Firmicutes, and Acidobacteria, were increased during AD events. Next, we performed a Metastats analysis to identify the phyla displaying significant differences between AD and non-AD periods (Table 2)^24^. Our data revealed a significant decrease in proportion (by 22.42%) of the predominant phylum, Proteobacteria, in AD samples. Simultaneously, Actinobacteria, Acidobacteria, Gemmatimonadetes, and Chloroflexi were increased 1.9, 2.2, 2.8, and 3.2 fold, respectively, in AD, compared to non-AD samples. In particular, the phylum Actinobacteria displayed the highest proportion (21.53%) except Proteobacteria during the AD period, consistent with previous data obtained in December 2014 in Seoul, Korea (manuscript under submission).

### Analysis of the bacterial community during AD and non-AD periods at the genus and species levels

To elucidate additional alterations in the airborne bacterial community during AD events, we analyzed the classification data at the genus and species levels (Tables 3 and 4). To this end, we focused on selected genera and species representing >0.5% of the total community. Analysis at the genus level revealed significant differences in the proportions of 21 genera, compared to non-AD samples. Moreover, all three genera belonging to the phylum Proteobacteria were decreased in AD samples. In particular, the proportions of *Escherichia-Shigella* and *Pseudomonas* were decreased by 89.8% and 52%, respectively. Although the proportion of genera showing increased abundance were relatively low within the entire classified bacterial community, most genera displayed a two to four fold increase in AD events, compared to non-AD periods. In particular, the proportion of the genus *Geodermatophilus* belonging to the phylum Actinobacteria was 6.42 times higher in AD than non-AD samples. Additionally, genera *Microvirga*, *Methylobacterium*, *Rubellimicrobium*, *Gemmatimonas* and *Modestobacter* presented relatively high proportions during AD events. In contrast to the Metastats analysis at the phylum level, the genus *Bacillus* belonging to the phylum Firmicutes was increased 3.93-fold in AD samples, constituting the highest proportion of significantly altered genera during AD events (from 1.9% to 7.6%). For analysis of the bacterial community at the species level, closely related species after classification of pyrosequencing data were selected based on the SILVA database. As shown in Table 4, among the species showing significant differences, *Bacillus circulans* displayed the highest increase in quantity (8.84-fold) and proportion (3.14%) in AD samples, followed by *Methylobacterium iners*, *Sphingomonas starnbergensis*, *Micrococcus terreus*, *Rubellimicrobium roseum*, and *Rubellimicrobium aerolatum*. In contrast, the proportions of *Escherichia coli*, *Escherichia fergusonii*, *Sphingomonas echinoides*, and *Sphingomonas oligophenolica* were decreased by ~90%. Despite slightly inaccurate classification at the species level (since the results were obtained using a limited length of the complete 16S rRNA gene sequence, taken as a whole), the genera *Bacillus* belonging to the phylum Firmicutes, *Escherichia* belonging to the phylum Proteobacteria, and *Modestobacter* belonging to the phylum Actinobacteria were clearly distinguished among the airborne bacterial communities during AD and non-AD periods.

## Discussion

In this study, we confirmed an increase in the total bacterial levels and community diversity in the airborne environment during the AD events in Korea. Moreover, the bacterial population was clearly distinguishable between AD and non-AD periods. Since AD events represent a natural phenomenon of transportation of dust particles from their region of origin and dust contains various microorganisms, we expected the samples collected during AD events to include newly transported, together with pre-existing microorganisms. As predicted, we obtained significantly higher values of diversity indices, including OTUs and Chao1, in AD samples, compared with non-AD samples, consistent with previous findings on dust particles^25^,^26^,^27^. Further examination of inverse Simpson index, which presents a richness feature with evenness diversity, confirmed that the evenness diversity index does not distinguish between AD and non-AD samples, in contrast to OTU results and Chao1 index, indicative of richness diversity. A previous report by Jeon *et al.*^27^ also showed that the Chao1 value of AD samples was 2.6 times higher than that of non-AD samples, while evenness values of each sample were determined as 0.851 and 0.887, respectively. Since evenness diversity reflects the number as well as proportion of specific OTUs, it is not surprising that this index is not altered by AD events. An *et al.*^28^ reported that richness values for control and AD samples are not correlated, while Mazar and co-workers proposed that the results are attributable to different sampling methods^26^. With the method of sampling, various conditions, including region and period of sampling, an origin of AD event, and meteorological conditions, may affect the biological characteristics of AD and non-AD samples and therefore consider for comparison. Therefore, it is important to clarify the features of AD events from various viewpoints and accumulate biological information consistently to obtain an overall picture of microbial alterations.

The airborne bacterial populations between AD events and non-AD periods were clearly distinguished using NMDS plot and phylogenetic tree analysis. In our experiments, PM10 concentration and 16S rRNA gene copy number, which were significantly correlated with AD events (Fig. 2), but not temperature, wind speed, and humidity, contributed to different bacterial community structures, suggesting that the differences between AD and non-AD samples are attributable to newly transported dust particles containing microorganisms. Several geochemical elements, including SO_2_ and NO_2_, have been correlated with PM10 concentration^29^. However, our data, in conjunction with other previous studies^30^,^31^ indicate that geochemical elements, including SO_2_, NO_2_, CO and O_3_, are not correlated with PM10 concentration, and do not differ significantly between AD and non-AD conditions during the period of sampling (independent t-test, P-values for SO_2_, NO_2_, CO, and O_3_ were 0.1918, 0.4348, 0.2143, and 0.9675, respectively., Supplementary Table S2). Information on airborne bacterial environments, together with geochemical and geophysical elements, may be valuable in elucidating the characteristics of dust particles between AD events and non-AD periods.

Finally, based on analysis of airborne bacterial communities from both AD and non-AD samples, we established that among the significantly altered genera, *Bacillus* and *Modestobacter* belonging to the phyla Firmicutes and Actinobacteria, respectively, had the highest proportions during AD events between February and March 2015. Consistent with our findings, increased proportions of Firmicutes and Actinobacteria in AD samples have been reported in previous studies^19^,^32^,^33^. The genus *Bacillus* has been isolated from soil, sediment, or sewage, and produces endospores, which may be present in the newly transported dust particles. *Bacillus circulans* is reported to cause sepsis, bacteremia, abscesses, and meningitis in humans^34^,^35^, suggesting that newly transported dust particles during AD events containing this microorganism could contribute to several human diseases. The species belonging to the genus *Modestobacter* is also found in soil or sediment, but does not produce endospores. However, common features of the genus *Modestobacter*, such as resistance to desiccation and irradiation^36^, facilitate survival in the desert, making it one of the major microorganism constituents of dust particles during AD events. At the time of writing the manuscript, four species of the genus *Modestobacter* had been identified. At present, there is no evidence to suggest a relationship between this bacterium and human disease. Interestingly, the proportion of the genus *Escherichia-Shigella* was largely decreased in AD samples (11.3% to 1.2%), consistent with results from a previous study^28^. Yamaguchi *et al.* also reported a relatively low proportion of the class *Gammaproteobacteria* containing the genus *Escherichia-Shigella* in Asian dust samples collected from Japan as well as its regions of origin, such as the Taklamakan and Gobi deserts^32^. Based on the 16S rRNA gene copy number results, which revealed that several hundred to thousand times more bacteria are present in AD samples (Fig. 2), we propose that the genus is rarely transported from its origin and present at a relatively low proportion in AD samples. The reasons underlying the low proportion of *Escherichia-Shigella* in dry or semi-dried regions remain to be established.

In conclusion, an in-depth investigation of the airborne bacterial community during AD events in this study confirmed that total amounts of bacteria in the airborne environment as well as their richness diversity are significantly altered, compared to the non-AD environment. Continuous monitoring of the characteristics of airborne bacterial community structures should be performed to obtain an overview of results that may provide important insights into the biological features of transported dust particles during AD events.

## Materials and Methods

### Collection of air samples

During AD and non-AD events between February to March 2015, dust samples were collected from the rooftop of Dongguk University in Goyang-si, South Korea (GPS: 37°40′41.7″ N, 126°48′25.6″ E). No buildings, such as commercial or industrial complexes, were present nearby that may have polluted or affected samples. For obtaining air samples, a high-volume air sampler (TE-5170, Tisch Environmental, Inc., USA) was used, and dust particles collected in cellulose filter paper at a flow rate of 700 L/min for 18 to 25 h. The collected dust particles in filter paper were stored at 4 °C until use. During the periods of sampling, nine days of AD events occurred and 11 non-AD days were selected for comparison (days of AD events: February 8, 22, and 23, March 1, 16, 17, 20, 21, and 22; non-AD days, February 5, 9, 12, and 26, March 2, 3, 10, 18, 19, 23, and 24). Airborne environmental information during this period (PM10 concentration, wind speed, humidity, temperature, and geochemical elements) was provided by Korea meteorological administration (KMA).

### DNA extraction and pyrosequencing

For genomic DNA extraction, 10 ml PBS was used to suspend collected dust particles on a 20 × 100 mm membrane. After resuspension, dust particles containing microorganisms were precipitated via centrifugation at 10,000 g for 1 min. Bacterial cells were lysed with DNA extraction buffer (100 mM Tris-Cl, pH 8.0; 100 mM sodium-EDTA, pH 8.0; 100 mM sodium phosphate, pH 8.0; 1.5 M NaCl; 1% CTAB) and 1 mg of proteinase K, followed by incubation at 37 °C for 30 min and subsequent addition of 2% SDS. After incubation at 65 °C for 30 min, cell debris and dust particles were precipitated via centrifugation. The supernatant fractions were mixed with an equal volume of chloroform and centrifuged. The upper layer was mixed with 70% v/v isopropanol and genomic DNA precipitated. After washing with 70% ethanol, the genomic DNA pellet was dried and resuspended with distilled water. For preparation of pyrosequencing samples, the 16S rRNA gene region was amplified with a set of primers containing the fourth and fifth hypervariable regions, according to a previous report^37^. The forward primer sequence was 517 F of the 16S rRNA gene (GCCAGCAGCCGCGGTAAT) and the reverse primer was 896 R (CCGTACTCCCCAGGCGG). For pyrosequencing, the forward and reverse primers were conjugated with adaptor sequences and multiplex identifier tag sequences provided by the manufacturer (Macrogen, Korea). Amplification of 16S rRNA gene was performed as follows: one cycle of pre-denaturation at 95 °C for 3 min, 35 cycles of denaturation at 95 °C for 3 s, and annealing and extension at 64 °C for 15 s. After amplification and purification (GeneAll Biotechnology, Korea), equal amounts (30 ng) of each PCR product were collected in a tube, and pyrosequencing performed with the GS FLX system (Roche, Switzerland).

### Quantification of 16S rRNA gene copy number

To determine the 16S rRNA gene copy number of each sample, *E. coli* strain ATCC 11775 was used to prepare a standard curve. Briefly, serially diluted *E.coli* strains were inoculated onto tryptone soy agar plates followed by counting growth colonies, and real-time PCR performed using an equal amount of *E. coli* as the template with the following primer set and amplification conditions: forward primer 517 F (GCCAGCAGCCGCGGTAAT), reverse primer 805 R (GACTACCAGGGTATCTAATCC), one cycle of pre-denaturation at 95 °C for 10 min, 30 cycles of denaturation at 95 °C for 3 sec, and annealing and extension at 60 °C for 15 sec. With the result of counted colonies, a standard curve was constructed using the threshold cycles of real-time PCR results. Based on the standard curve, the 16S rRNA gene copy number was assessed from real-time PCR data obtained using the samples collected as template. Real-time PCR was performed using the same conditions and primer sets as above.

### Data analysis

Pyrosequencing results were analyzed using standard Mothur software^22^. Briefly, all low-quality sequences, including small fragments (<200 bp) and chimeric sequences, were removed using Mother and UCHIME software^38^, respectively. Various sequence numbers (2,445 to 14,771) were obtained after trimming low-quality data of pyrosequencing results and 2,445 sequences of subsamples randomly selected for normalization of read numbers. After trimming the sequences, the SILVA database was used for classification of the bacterial community^23^, and statistical analysis of the classified results performed using Metastats software^24^. Following classification, mitochondria, chloroplast, archaea and eukaryote-associated sequences were removed. For diversity analysis, we used a phylotype analysis method based on classified taxonomic information rather than cutoff values (using Mothur command, phylotype), since several sequences classified into the same taxonomy presented different OTUs using a distance-based cutoff method. The airborne bacterial community was analyzed at the phylum, genus, and species levels using taxonomic information, and bacterial diversity indices and NMDS plots computed using phylotype results at the species level. The correlation coefficient between NMDS plot and meteorological elements was calculated via metadata analysis with Mothur software. Statistical values, including Pearson and Spearman correlation coefficients and independent t-tests, were calculated using R software with the Rcmd package^39^.

## Additional Information

**How to cite this article**: Cha, S. *et al.* Alterations in the airborne bacterial community during Asian dust events occurring between February and March 2015 in South Korea. *Sci. Rep.* **6**, 37271; doi: 10.1038/srep37271 (2016).

**Publisher’s note**: Springer Nature remains neutral with regard to jurisdictional claims in published maps and institutional affiliations.

## Supplementary Material

Supplementary Information

## Figures and Tables

**Table 1:** Calculation of the correlation coefficient between the nonmetric multidimensional scaling plot and experimental or environmental factors.

Factors	Correlation coefficient with axis 1	P-value	Correlation coefficient with axis 2	P-value
16S rRNA gene copy number	−0.55085	0.006918	0.094531	0.679398
PM10 concentration	−0.46113	0.029708	−0.01391	0.461337
Temperature	−0.18942	0.403281	0.08213	0.719742
Wind speed	−0.12898	0.571823	−0.18204	0.422291
Humidity	0.119897	0.599482	−0.07429	0.745606

P-values < 0.05 mean that the indicated factor is correlated with the direction of plot axis. 16S rRNA gene copy number and PM10 concentration, but not other factors, were significantly correlated with the negative direction along axis1 of the plots.

**Table 2:** Metastats analysis at the phylum level.

Phylum	AD			Non-AD			P-value^b^	Ratio of AD/non-AD
	Mean^a^	Variance	Standard error	Mean^a^	Variance	Standard error		
Proteobacteria	53.53	206.87	4.79	69.00	233.24	4.60	0.022	0.776
Actinobacteria	21.53	52.12	2.41	11.23	81.06	2.71	0.01	1.917
Acidobacteria	1.61	0.94	0.32	0.74	0.28	0.16	0.028	2.167
Gemmatimonadetes	1.60	1.53	0.41	0.57	0.44	0.20	0.038	2.793
Chloroflexi	0.66	0.18	0.14	0.21	0.06	0.07	0.011	3.222

The table contains data on significantly different phyla (P-value < 0.05) with a proportion greater than 0.5% of the total bacterial community. ^a^Mean values signify the average proportion of the phylum. ^b^P-values < 0.05 indicate significant differences in proportion between the two groups (AD events and non-AD days).

**Table 3:** Metastats analysis at the genus level.

Genus	AD			Non-AD			P-value^b^	Ratio of AD/non-AD
	Mean^a^	Variance	Standard error	Mean^a^	Variance	Standard error		
*Pseudomonas*	1.791	3.110	0.588	3.733	6.240	0.753	0.041	0.480
*Escherichia-Shigella*	1.158	2.210	0.496	11.374	644.740	7.656	0.026	0.102
*Alicycliphilus*	0.063	0.010	0.025	0.535	0.580	0.230	0.003	0.117
*Devosia*	0.540	0.040	0.066	0.268	0.110	0.102	0.049	2.015
*Paracoccus*	0.725	0.170	0.138	0.352	0.100	0.098	0.033	2.060
*Microvirga*	1.724	1.370	0.390	0.442	0.360	0.182	0.004	3.900
*Methylobacterium*	1.565	0.230	0.158	0.835	0.240	0.148	0.003	1.874
*Belnapia*	0.681	0.350	0.197	0.161	0.040	0.057	0.003	4.223
*Rubellimicrobium*	1.846	1.180	0.363	0.417	0.450	0.202	0.002	4.425
*Gemmatimonas*	1.555	1.440	0.400	0.552	0.420	0.195	0.031	2.817
*Bacillus*	7.632	113.460	3.551	1.939	1.890	0.414	0.005	3.937
*Sphaerobacter*	0.596	0.160	0.134	0.160	0.040	0.060	0.007	3.726
*Blastococcus*	1.081	0.410	0.213	0.486	0.230	0.144	0.028	2.225
*Solirubrobacter*	0.615	0.250	0.168	0.221	0.040	0.061	0.019	2.779
*Pseudonocardia*	0.504	0.210	0.153	0.144	0.030	0.050	0.019	3.509
*Nocardioides*	0.991	0.300	0.183	0.404	0.230	0.144	0.015	2.455
*Cellulomonas*	0.749	0.220	0.156	0.254	0.130	0.107	0.015	2.949
*Micrococcus*	0.542	0.040	0.068	0.243	0.080	0.087	0.014	2.233
*Modestobacter*	3.006	2.790	0.557	1.121	1.400	0.357	0.007	2.683
*Geodermatophilus*	0.979	0.790	0.296	0.152	0.030	0.049	0.003	6.429
*Blastocatella*	0.713	0.300	0.183	0.222	0.040	0.062	0.009	3.207

The table contains data on significantly different genera (P-value < 0.05) with a proportion greater than 0.5% of the total bacterial community. ^a^Mean values signify the average proportion of the genera. ^b^P-values < 0.05 indicate significant differences in proportion between the two groups (AD events and non-AD days).

**Table 4:** Metastats analysis at the species level.

Species	AD			Non-AD			P-value^b^	Ratio of AD/non-AD
	Mean^a^	Variance	Standard error	Mean^a^	Variance	Standard error		
*Methylobacterium iners*	0.677	0.130	0.119	0.099	0.020	0.039	0.001	6.830
*Sphingomonas starnbergensis*	1.033	0.830	0.304	0.189	0.030	0.051	0.004	5.475
*Rubellimicrobium roseum*	0.935	0.350	0.196	0.204	0.120	0.103	0.009	4.590
*Rubellimicrobium aerolatum*	0.888	0.310	0.184	0.195	0.100	0.093	0.003	4.549
*Sphingomonas melonis*	0.845	0.390	0.207	0.190	0.040	0.057	0.003	4.445
*Belnapia soli*	0.542	0.320	0.190	0.146	0.030	0.054	0.027	3.719
*Sphingomonas ginsengisoli*	0.886	0.630	0.265	0.241	0.070	0.079	0.026	3.670
*Sphingomonas oryziterrae*	0.820	0.390	0.208	0.224	0.080	0.086	0.011	3.665
*Sphingomonas kaistensis*	1.006	0.650	0.268	0.307	0.120	0.103	0.022	3.274
*Sphingomonas astaxanthinifaciens*	0.539	0.190	0.147	0.168	0.050	0.069	0.020	3.208
*Sphingomonas jaspsi*	1.396	0.800	0.298	0.493	0.310	0.167	0.012	2.833
*Sphingomonas echinoides*	0.491	0.430	0.219	4.302	19.780	1.341	0.002	0.114
*Sphingomonas oligophenolica*	0.380	0.220	0.157	3.381	12.280	1.057	0.001	0.112
*Acidovorax caeni*	0.882	0.680	0.274	0.198	0.090	0.089	0.035	4.451
*Comamonas denitrificans*	0.156	0.040	0.063	0.625	0.290	0.162	0.008	0.250
*Alicycliphilus denitrificans K601*	0.063	0.010	0.025	0.535	0.580	0.230	0.004	0.117
*Escherichia coli*	1.069	1.890	0.458	10.447	547.130	7.053	0.022	0.102
*Escherichia fergusonii*	0.041	0.000	0.016	0.578	1.540	0.374	0.017	0.070
*Gemmatimonas aurantiaca*	1.411	1.160	0.359	0.499	0.320	0.171	0.044	2.828
*Bacillus circulans*	3.138	30.100	1.829	0.355	0.190	0.130	0.005	8.838
*Micrococcus terreus*	0.500	0.040	0.063	0.103	0.030	0.052	0.001	4.869
*Solirubrobacter soli*	0.509	0.200	0.149	0.153	0.030	0.054	0.032	3.329
*Modestobacter marinus*	1.929	1.090	0.348	0.737	0.620	0.237	0.014	2.618
*Blastocatella fastidiosa*	0.713	0.300	0.183	0.222	0.040	0.062	0.012	3.207

The table contains data on significantly different species (P-value < 0.05) with a proportion greater than 0.5% of the total bacterial community. ^a^Mean values signify the average proportion of the species. ^b^P-values < 0.05 indicate significant differences in proportion between the two groups (AD events and non-AD days).

## References

[b1] Lei Y. C., Chan C. C., Wang P. Y., Lee C. T. & Cheng T. J. Effects of Asian dust event particles on inflammation markers in peripheral blood and bronchoalveolar lavage in pulmonary hypertensive rats. Environ Res 95, 71–76, doi: 10.1016/S0013-9351(03)00136-1 (2004). 15068932

[b2] Onishi K. *et al.* Atmospheric transport route determines components of Asian dust and health effects in Japan. Atmos Environ 49, 94–102, doi: 10.1016/j.atmosenv.2011.12.018 (2012).

[b3] Liu C. M., Young C. Y. & Lee Y. C. Influence of Asian dust storms on air quality in Taiwan. Science of the Total Environment 368, 884–897, doi: 10.1016/j.scitotenv.2006.03.039 (2006). 16674998

[b4] Buseck P. R. & Posfai M. Airborne minerals and related aerosol particles: Effects on climate and the environment. P Natl Acad Sci USA 96, 3372–3379, doi: 10.1073/pnas.96.7.3372 (1999). PMC34277 10097046

[b5] Chun Y. S., Boo K. O., Kim J., Park S. U. & Lee M. Synopsis, transport, and physical characteristics of Asian dust in Korea. J Geophys Res-Atmos 106, 18461–18469, doi: 10.1029/2001jd900184 (2001).

[b6] Han Y. X., Fang X. M., Zhao T. L. & Kang S. C. Long range trans-Pacific transport and deposition of Asian dust aerosols. J Environ Sci-China 20, 424–428, doi: 10.1016/S1001-0742(08)62074-4 (2008). 18575126

[b7] Kim K. H. & Kim M. Y. The effects of Asian Dust on particulate matter fractionation in Seoul, Korea during spring 2001. Chemosphere 51, 707–721, doi: 10.1016/S0045-3565(03)00036-5 (2003). 12668030

[b8] Youngsin C. & Lim J. Y. The recent characteristics of Asian dust and haze events in Seoul, Korea. Meteorol Atmos Phys 87, 143–152, doi: 10.1007/s00703-003-0067-2 (2004).

[b9] Onishi K., Otani S., Yoshida A., Mu H. S. & Kurozawa Y. Adverse health effects of Asian dust particles and heavy metals in Japan. Asia-Pac J Public He 27, 1719–1726, doi: 10.1177/1010539511428667 (2015). 22865718

[b10] Kishikawa R. T. *et al.* Effects of Asian dust and spherical particles exposure on human health and allergic symptom, Fukuoka, Japan. J Allergy Clin Immun 129, 59, doi: 10.1016/j.jaci.2011.12.717 (2012).

[b11] Hong Y. C. *et al.* Asian dust storm and pulmonary function of school children in Seoul. Sci Total Environ 408, 754–759, doi: 10.1016/j.scitotenv.2009.11.015 (2010). 19939437

[b12] Lee J. T., Son J. Y. & Cho Y. S. A comparison of mortality related to urban air particles between periods with Asian dust days and without Asian dust days in Seoul, Korea, 2000–2004. Environmental Research 105, 409–413, doi: 10.1016/j.envres.2007.06.004 (2007). 17659273

[b13] He M. *et al.* Differences in allergic inflammatory responses between urban PM2.5 and fine particle derived from desert-dust in murine lungs. Toxicol Appl Pharmacol 297, 41–55, doi: 10.1016/j.taap.2016.02.017 (2016). 26917405

[b14] Capasso L., Longhin E., Caloni F., Camatini M. & Gualtieri M. Synergistic inflammatory effect of PM10 with mycotoxin deoxynivalenol on human lung epithelial cells. Toxicon 104, 65–72, doi: 10.1016/j.toxicon.2015.08.008 (2015). 26263889

[b15] Huttunen K. *et al.* Inflammatory potential in relation to the microbial content of settled dust samples collected from moisture-damaged and reference schools: results of HITEA study. Indoor Air 26, 380–390, doi: 10.1111/ina.12223 (2015). 25967114

[b16] Ortiz-Martinez M. G., Rodriguez-Cotto R. I., Ortiz-Rivera M. A., Pluguez-Turull C. W. & Jimenez-Velez B. D. Linking endotoxins, African dust PM10 and asthma in an urban and rural environment of Puerto Rico. Mediat Inflamm, doi: 10.1155/2015/784212 (2015). PMC4670654 26681839

[b17] He M. *et al.* Airborne Asian sand dust enhances murine lung eosinophilia. Inhal Toxicol 22, 1012–1025, doi: 10.3109/08958378.2010.510151 (2010). 20849355

[b18] Nishimura Y. *et al.* Similarity of bacterial community structure between Asian aust and its sources determined by rRNA gene-targeted approaches. Microbes Environ 25, 22–27, doi: 10.1264/jsme2.ME09166 (2010). 21576848

[b19] Yamaguchi N. *et al.* Changes in the airborne bacterial community in outdoor environments following Asian dust events. Microbes Environ 29, 82–88, doi: 10.1264/jsme2.ME13080 (2014). 24553107 PMC4041233

[b20] Lee S., Choi B., Yi S. M. & Go G. Characterization of microbial community during Asian dust events in Korea. Sci Total Environ 407, 5308–5314, doi: 10.1016/j.scitotenv.2009.06.052 (2009). 19631361

[b21] Kenzaka T. *et al.* Soil microbial community structure in an Asian dust source region (Loess Plateau). Microbe Environ 25, 53–57, doi: 10.1264/jsme2.ME09164 (2010). 21576854

[b22] Schloss P. D. *et al.* Introducing mothur: open-source, platform-independent, community-supported software for describing and comparing microbial communities. Appl Environ Microb 75, 7537–7541, doi: 10.1128/Aem.01541-09 (2009). PMC2786419 19801464

[b23] Quast C. *et al.* The SILVA ribosomal RNA gene database project: improved data processing and web-based tools. Nucleic Acids Res 41, 590–596, doi: 10.1093/nar/gks1219 (2013). PMC3531112 23193283

[b24] White J. R., Nagarajan N. & Pop M. Statistical methods for detecting differentially abundant features in clinical metagenomic samples. Plos Comput Biol 5, doi: 10.1371/journal.pcbi.1000352 (2009). PMC2661018 19360128

[b25] Barberán A. *et al.* Continental-scale distributions of dust-associated bacteria and fungi. P Natl Acad Sci USA 112, 5756–5761, doi: 10.1073/pnas.1420815112 (2015). PMC4426398 25902536

[b26] Mazar Y., Cytryn E., Erel Y. & Rudich Y. Effect of dust storms on the atmospheric microbiome in the Eastern Mediterranean. Environ Sci Technol 50, 4194–4202, doi: 10.1021/acs.est.5b06348 (2016). 27001166

[b27] Jeon E. M. *et al.* Impact of Asian dust events on airborne bacterial community assessed by molecular analyses. Atmos Environ 45, 4313–4321, doi: 10.1016/j.atmosenv.2010.11.054 (2011).

[b28] An S., Sin H. H. & Dubow M. S. Modification of atmospheric sand-associated bacterial communities during Asian sandstorms in China and South Korea. Heredity 114, 460–467, doi: 10.1016/j.atmosenv.2010.11.054 (2015). 25388140 PMC4815509

[b29] Fu Q. *et al.* Source, long-range transport, and characteristics of a heavy dust pollution event in Shanghai. J Geophys res 115, doi: 10.1029/2009JD013208 (2010).

[b30] Chan C. C., Chuang K. J., Chien L. C., Chen W. J. & Chang W. T. Urban air pollution and emergency admissions for cerebrovascular diseases in Taipei, Taiwan. Eur Heart J 27, 1238–1244, doi: 10.1093/eurheartj/ehi835 (2006). 16537554

[b31] Woodruff T. J., Darrow L. A. & Parker J. D. Air pollution and postneonatal infant mortality in the United States, 1999–2002. Environ Health Perspect 116, 110–115, doi: 10.1289/ehp.10370 (2008). 18197308 PMC2199284

[b32] Yamaguchi N., Ichijo T., Sakotani A., Baba T. & Nasu M. Global dispersion of bacterial cells on Asian dust. Sci Rep 2, 525, doi: 10.1038/srep00525 (2012). 22826803 PMC3401963

[b33] Yamaguchi N. *et al.* Abundance and community structure of bacteria on Asian dust particles collected in Beijing, China, during the Asian dust season. Biol Pharm Bull 39, 68–77, doi: 10.1248/bpb.b15-00573 (2016). 26725429

[b34] Alebouyeh M. *et al.* Fatal sepsis by *Bacillus circulans* in an immunocompromised patient. *Iran* J Microbiol 3, 156–158 (2011). PMC3279812 22347600

[b35] Logan N. A., Old D. C. & Dick H. M. Isolation of *Bacillus circulans* from a wound infection. J Clin Pathol 38, 838–839 (1985). 4019805 10.1136/jcp.38.7.838 PMC499315

[b36] Gtari M. *et al.* Contrasted resistance of stone-dwelling Geodermatophilaceae species to stresses known to give rise to reactive oxygen species. FEMS Microbiol Ecol 80, 566–577, doi: 10.1111/j.1574-6941.2012.01320.x (2012). 22296311

[b37] Vasileiadis S. *et al.* Soil bacterial diversity screening using single 16S rRNA gene V regions coupled with multi-million read generating sequencing technologies. Plos One 7, doi: 10.1371/journal.pone.0042671 (2012). PMC3412817 22880076

[b38] Edgar R. C., Haas B. J., Clemente J. C., Quince C. & Knight R. UCHIME improves sensitivity and speed of chimera detection. Bioinformatics 27, 2194–2200, doi: doi: 10.1093/bioinformatics/btr381 (2011). 21700674 PMC3150044

[b39] Fox J. The R Commander: A basic-statistics graphical user interface to R. J Stat Softw 14, doi: 10.18637/jss.v014.i09 (2005).

