# Supplementary material for: Alterations in the airborne bacterial community during Asian dust events occurring between February and March 2015 in South Korea
Source: Sci Rep. 2016 Nov 16;6:37271. doi: 10.1038/srep37271 (PMC5110963; doi:10.1038/srep37271)

Supplementary information

Title

Alteration in the airborne bacterial community during Asian dust events occurring between February and March 2015 in South Korea

Authors

Seho Cha, Dongwook Lee, Jun Hyeong Jang, Sora Lim, Dahye Yang, and Taegun Seo [*]

Affiliation

Department of Life Science, Dongguk University-Seoul, Goyang, 10326, South Korea

*Corresponding author

Supplementary Table S1. Data for pyrosequencing results and alpha diversity during the period of sampling.

Date	Total reads	Normalized Reads	OTUs	Chao1	Inversed Simpson
02/05/2015	2445	2445	173	214	12
02/08/2015 (AD)	4076	2445	733	1265	100
02/09/2015	4679	2445	458	729	28
02/12/2015	3363	2445	246	311	15
02/22/2015 (AD)	6358	2445	506	952	82
02/23/2015 (AD)	11321	2445	545	846	32
02/26/2015	14771	2445	146	248	7
03/01/2015 (AD)	5891	2445	328	404	43
03/02/2015	3008	2445	415	669	34
03/03/2015	2471	2445	564	1016	59
03/10/2015	5029	2445	68	116	2
03/16/2015 (AD)	4816	2445	394	514	52
03/17/2015 (AD)	5107	2445	540	708	121
03/18/2015	2886	2445	400	515	43
03/19/2015	3836	2445	733	1315	194
03/20/2015 (AD)	3649	2445	628	1254	130
03/21/2015 (AD)	5337	2445	710	1227	164
03/22/2015 (AD)	2785	2445	723	1279	102
03/23/2015	9473	2445	579	763	134
03/24/2015	10813	2445	170	205	23

Supplementary Table S2. Data for geochemical elements during the period of sampling. *ND, Not determined.

Date	SO_2 (ppm)	CO (ppm)	O_3 (ppm)	NO_2 (ppm)
02/05/2015	0.006842	0.573684	0.022947	0.023
02/08/2015 (AD)	0.005083	0.333333	0.021583	0.009625
02/09/2015	0.006316	0.610526	0.010444	0.0295
02/12/2015	0.004789	0.405263	0.021211	0.016579
02/22/2015 (AD)	0.003273	0.404545	0.018682	0.012545
02/23/2015 (AD)	0.004125	0.558333	0.017042	0.022375
02/26/2015	0.004318	0.295455	0.027318	0.007273
03/01/2015 (AD)	0.005364	0.427273	0.019818	0.021136
03/02/2015	0.006818	0.522727	0.013864	0.034909
03/03/2015	0.004182	0.286364	0.026955	0.006773
03/10/2015	0.005444	0.461111	0.017	0.022611
03/16/2015 (AD)	0.011944	0.922222	0.008167	0.071667
03/17/2015 (AD)	0.007111	0.638889	0.014056	0.035944
03/18/2015	0.004611	0.433333	0.024722	0.0175
03/19/2015	0.006389	0.7	0.012833	0.051722
03/20/2015 (AD)	0.011389	0.761111	0.0105	0.059444
03/21/2015 (AD)	0.0125	0.894444	0.029667	0.039056
03/22/2015 (AD)	0.00508	0.356	0.02968	0.01448
03/23/2015	ND*	ND*	ND*	ND*
03/24/2015	0.005833	0.461111	0.009333	0.0395

Supplementary figure S1. Construction of phylogenetic tree based on the ThetaYC algorithm at species level

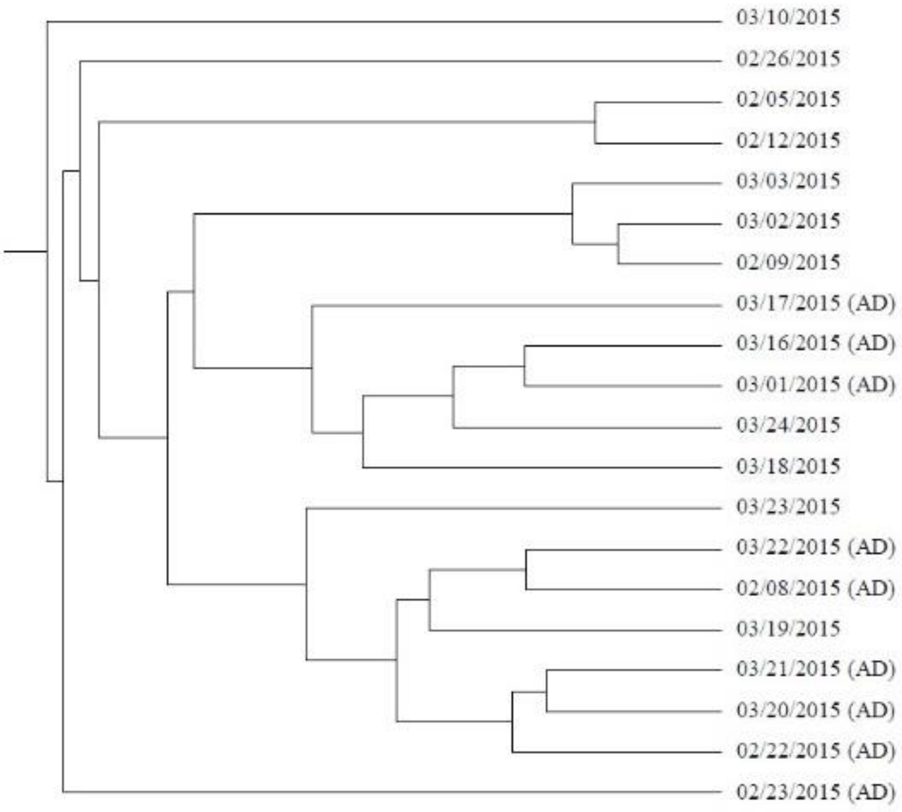

Supplement: Supplementary Information [file srep37271-s1.pdf]
